# PltRNAdb: Plant transfer RNA database

**DOI:** 10.1371/journal.pone.0268904

**Published:** 2022-05-23

**Authors:** Morad M. Mokhtar, Achraf EL Allali

**Affiliations:** African Genome Center, University Mohammed VI Polytechnic, Benguerir, Morocco; Youngstown State University, UNITED STATES

## Abstract

Transfer RNAs (tRNAs) are intermediate-sized non-coding RNAs found in all organisms that help translate messenger RNA into protein. Recently, the number of sequenced plant genomes has increased dramatically. The availability of this extensive data greatly accelerates the study of tRNAs on a large scale. Here, 8,768,261 scaffolds/chromosomes containing 229,093 giga-base pairs representing whole-genome sequences of 256 plant species were analyzed to identify tRNA genes. As a result, 331,242 nuclear, 3,216 chloroplast, and 1,467 mitochondrial tRNA genes were identified. The nuclear tRNA genes include 275,134 tRNAs decoding 20 standard amino acids, 1,325 suppressor tRNAs, 6,273 tRNAs with unknown isotypes, 48,475 predicted pseudogenes, and 37,873 tRNAs with introns. Efforts also extended to the creation of PltRNAdb (https://bioinformatics.um6p.ma/PltRNAdb/index.php), a data source for tRNA genes from 256 plant species. PltRNAdb website allows researchers to search, browse, visualize, BLAST, and download predicted tRNA genes. PltRNAdb will help improve our understanding of plant tRNAs and open the door to discovering the unknown regulatory roles of tRNAs in plant genomes.

## Introduction

Transfer RNAs (tRNAs) are intermediate-sized non-coding RNA genes discovered in all organisms that help in the translation of mRNA into protein [1]. tRNAs are found in all types of cells and organelles and are involved in several cellular processes, including viral replication, amino acid biosynthesis, and cell wall remodeling [2, 3]. In plants, tRNA undergoes a post-transcriptional process to obtain the mature form required for its function [4]. Recently, Hummel et al. [5] reported a variety of cell biological processes that are affected by the organization, expression, and modification of tRNA genes. These modifications are a source of novel biological functions of tRNAs in plants [6].

In the last decade, the number of sequenced plant genomes has increased with the advances in sequencing technology [7]. It is of critical importance to predict tRNA genes in sequenced genomes as continuous. Thanks to advances in bioinformatics, several tools have been developed to predict tRNA genes. These tools include tRNAscan-SE [8], ARAGORN [9], DOGMA [10], ARWEN [11], MiTFi [12], TFAM [13], tRNAfinder [14], and SPLITS [15]. tRNAscan-SE [8] is the most widely used tool for detecting and annotating tRNA genes in sequenced genomes. Both computational prediction tools and tRNA databases are used to identify tRNA genes of specific plant species. There are several tRNA databases such as tRNAdb http://trna.bioinf.uni-leipzig.de/DataOutput/ [16], GtRNAdb http://gtrnadb.ucsc.edu/ [17], tRNADB-CE http://trna.ie.niigata-u.ac.jp/cgi-bin/trnadb/index.cgi [18], and PlantRNA http://plantrna.ibmp.cnrs.fr/ [19]. Unfortunately, these databases are out of date as they do not use recently sequenced plant genomes (Table 1).

**Table 1:** Comparison between PltRNAdb (current database) and other tRNA databases.

Database	No. of plants	tRNA genes	Database Link
**PltRNAdb (Current database)**	256	337555	https://bioinformatics.um6p.ma/PltRNAdb/index.php
**PtRNAdb** [20]	106	113849	www.nipgr.ac.in/PtRNAdb
**tRNAdb** [16]	58	702	http://trna.bioinf.uni-leipzig.de/DataOutput/
**PlantRNA** [19]	47	66686	http://plantrna.ibmp.cnrs.fr/
**GtRNAdb** [17]	15	30061	http://gtrnadb.ucsc.edu/
**tRNADB-CE** [18]	2	1352	http://trna.ie.niigata-u.ac.jp/cgi-bin/trnadb/index.cgi

Due to the increasing number of plant genomes, we have developed PltRNAdb, a freely available database of tRNA genes from 256 plant species. The PltRNAdb database contains the details of identified tRNAs in the nuclear genome and its available organellar genomes as follows: 1) tRNA sequences, 2) tRNA secondary structure visualization, 3) tRNAs upstream and downstream sequences, 4) tRNAs with introns, 5) tRNAs decoding 20 standard amino acids, 6) possible suppressor tRNAs, 7) tRNAs with unknown isotypes, 8) predicted tRNA pseudo-genes. We hope that by pooling such extensive data into one database, we can improve our understanding of plant tRNAs and open the door to discovering the unknown regulatory roles of tRNAs in plant genomes.

## Materials and methods

### Genomic data

We retrieved FASTA files of sequenced and annotated nuclear genomes for 256 plant species from the NCBI database (https://www.ncbi.nlm.nih.gov/). In addition, we retrieved the available organellar genomes of those species, including 100 chloroplast and 52 mitochondrial genomes. The 256 plant genomes include 229 Streptophyta, 24 Chlorophyta, and 3 Rhodophyta (Table 2). The details of the studied species are listed in S1 Table, including plant name, NCBI taxid, assembly type and level, sequence representation and coverage, and sequence category and accession numbers.

**Table 2:** List of plant family names and total number of species used in the present study.

Streptophyta						Chlorophyta	
Family name	Species	Family name	Species	Family name	Species	Family name	Species
Malvaceae	23	Nymphaeaceae	2	Moraceae	1	Chlamydomonadaceae	4
Fabaceae	22	Myrtaceae	2	Lythraceae	1	Volvocaceae	3
Poaceae	20	Marchantiaceae	2	Lentibulariaceae	1	Bathycoccaceae	3
Brassicaceae	16	Lamiaceae	2	Lauraceae	1	Chlorellaceae	3
Solanaceae	13	Fagaceae	2	Klebsormidiaceae	1	Mamiellaceae	2
Rosaceae	11	Dioscoreaceae	2	Gesneriaceae	1	Selenastraceae	2
Salicaceae	8	Arecaceae	2	Funariaceae	1	Dunaliellaceae	1
Cucurbitaceae	8	Apiaceae	2	Ditrichaceae	1	Pycnococcaceae	1
Asteraceae	6	Anacardiaceae	2	Cyperaceae	1	Chloropicaceae	1
Orchidaceae	4	Zosteraceae	1	Cleomaceae	1	Tetrabaenaceae	1
Euphorbiaceae	4	Zingiberaceae	1	Circaeasteraceae	1	Astrephomenaceae	1
Ericaceae	4	Vitaceae	1	Characeae	1	Others	2
Convolvulaceae	4	Trochodendraceae	1	Cephalotaceae	1		
Rutaceae	3	Theaceae	1	Celastraceae	1		
Rubiaceae	3	Selaginellaceae	1	Caricaceae	1	**Rhodophyta**	
Ranunculaceae	3	Scrophulariaceae	1	Bromeliaceae	1	Cyanidiaceae	2
Musaceae	3	Sapindaceae	1	Boraginaceae	1	Gigartinaceae	1
Juglandaceae	3	Pteridaceae	1	Bignoniaceae	1		
Chenopodiaceae	3	Phrymaceae	1	Betulaceae	1		
Cannabaceae	3	Pedaliaceae	1	Asparagaceae	1		
Rhamnaceae	2	Oleaceae	1	Aristolochiaceae	1		
Proteaceae	2	Nyssaceae	1	Araceae	1		
Papaveraceae	2	Nelumbonaceae	1	Amborellaceae	1		
Orobanchaceae	2	Myricaceae	1	Actinidiaceae	1		

### Prediction of tRNA genes

tRNAscan-SE v.2.0.9 [21] was used in the present study for the prediction of tRNAs in the studied plant genomes. For nuclear genomes, the parameters were set to: Search Mode: Eukaryotic, Searching with: Infernal first pass, Isotype-specific model scan: yes, Covariance model: TRNAinf-euk.cm, Infernal first pass cutoff score: 10, and Temporary directory: tmp. For chloroplast and mitochondrial genomes, the parameters were set as follows: Search Mode: (Organellar), Searching with: (Infernal single-pass; scan Maximum sensitivity mode), Covariance model: (TRNAinf-1415.cm), Cutoff score: (15).

### Database construction

**Fig 1:**
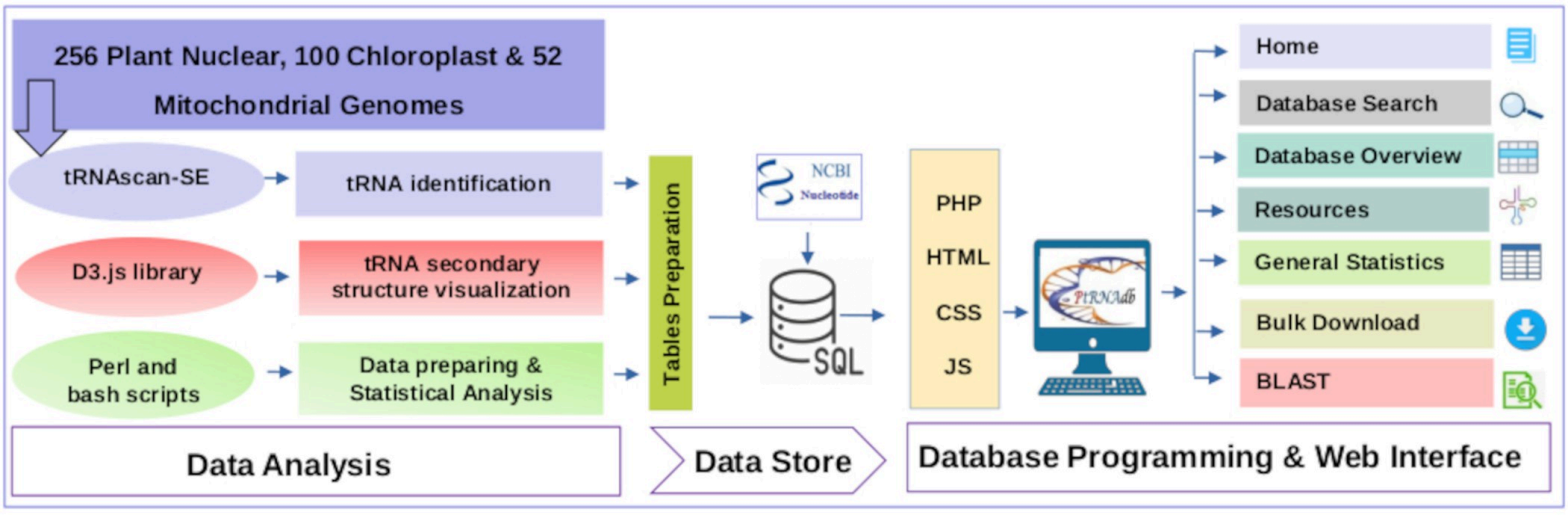
The workflow used in the identification of tRNA genes and the construction of the PltRNAdb database.

The PltRNAdb database was created using Apache 2.4.41, MySQL 8.0.27, PHP 7.4.3., Perl 5.30.0, Python 3.8.10, and the D3 library. The interactive web interface was designed using PHP, CSS, HTML, and JavaScript. The workflow for identifying the tRNA and creating the PltRNAdb is shown in (Fig 1). Data-Driven-Documents (D3.js, https://github.com/d3) was implemented in our PltRNAdb to visualize the secondary structure of all predicted tRNAs.

## Results and discussion

Recently, the number of sequenced plant genomes has increased due to advances in genome sequencing. This large number of sequenced genomes requires bioinformatics tools to extract various features and make them available in various databases. For plants, almost half of the sequenced genomes have not yet been subjected to tRNA prediction using the available tools [8–15]. Consequently, the recently sequenced plant genomes are not included in the current tRNA databases [16–19].

### Prediction tRNAs

**Fig 2:**
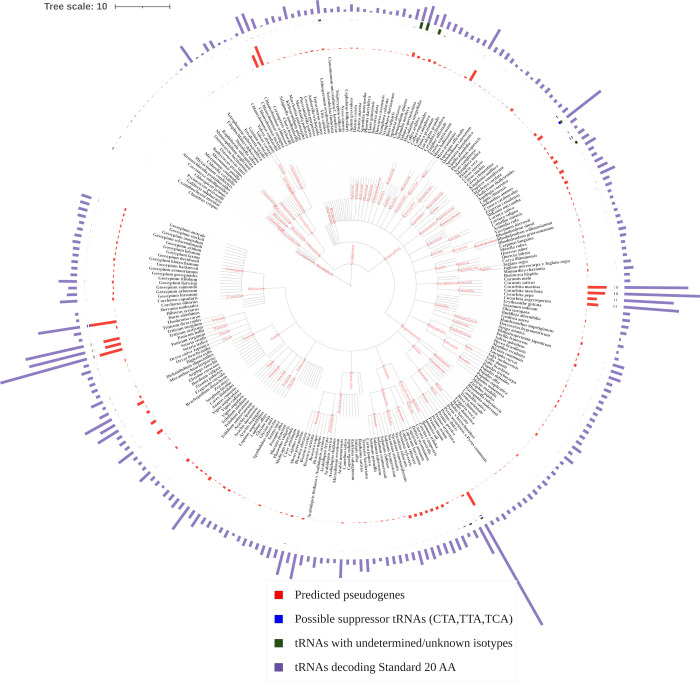
The bar chart represents the comparisons of nuclear genomes of the studied plant species. The inner circle represents 256 plant species, and the outer circle includes tRNA decoding the standard 20 amino acids (purple color), predicted pseudogenes (red color), possible suppressor tRNAs (blue color), and tRNA with undetermined isotypes (green color).

Here, 8,768,261 scaffolds/chromosomes with a total length of 229,093 giga base pairs representing nuclear, chloroplast and mitochondrial genome sequences of the studied plant species were analyzed to identify the tRNA genes. As a result, 331,242, 3,216, and 1,467 tRNA genes were identified from nuclear, chloroplast and mitochondrial genomes, respectively (Table 3). Fig 2 shows bar charts for the total number of nuclear tRNAs decoding 20 standard amino acids, suppressor tRNAs, tRNAs with unknown isotypes, and predicted pseudo-genes for each genome.

**Table 3:** Total numbers of tRNAs, the tRNAs decoding 20 standard amino acids, suppressor tRNAs (TTA, CTA, TCA), tRNAs with unknown isotypes, predicted pseudo-genes, and tRNAs with introns for the nuclear, chloroplast and mitochondrial genomes.

	Total tRNAs	tRNAs decoding Standard 20 AA	Suppressor tRNAs	tRNAs with unknown isotypes	Predicted pseudo-genes	tRNAs with introns
**Nuclear**	331242	275134	1325	6273	48475	37873
**Mitochondrial**	1467	1390	7	70	1	97
**Chloroplast**	3216	3200	1	2	13	299

To date, further efforts have been made to predict plant tRNA genes and make them available by building web databases. Several databases have been created using tRNAscan, including GtRNAdb [21], tRNAdb [16], tRNADB-CE [18], and PlantRNA [19]. GtRNAdb contains 30,061 predicted tRNA genes derived from 15 plant species, whereas tRNAdb contains 702 tRNA genes derived from 58 plant species. In addition, tRNADB-CE contains 1,352 tRNA genes derived from 2 plant species, while PlantRNA database contains 66,686 genes derived from 47 plant species. Table 4 compares our database with previously developed databases (GtRNAdb and PlantRNA). This comparison includes only the sequenced and annotated plant species (38 species) shared by the compared databases. The comparison includes the number of predicted tRNA genes and the number of tRNAs with introns. The species name, total number of predicted tRNA genes, and number of tRNAs with introns of 218 plant species available only through the current database were listed in S2 Table.

**Table 4:** Comparison between PltRNAdb (current database), GtRNAdb and PlantRNA databases based on the total number of tRNAs (Total) and the number of tRNAs with introns (WI).

Plant Species	PltRNAdb		GtRNAdb		PlantRNA	
	Total	WI	Total	WI	Total	WI
***Amborella trichopoda***	569	24	-	-	542	12
***Arabidopsis lyrata***	643	55	-	-	656	51
***Arabidopsis thaliana***	642	82	642	82	643	81
***Brachypodium distachyon***	625	30	625	30	626	26
***Brassica napus***	3053	226	3053	226	3053	163
***Brassica oleracea***	1338	94	-	-	1338	62
***Brassica rapa***	1254	94	-	-	1254	82
***Capsella rubella***	597	49	-	-	597	48
***Capsicum annuum***	1741	53	-	-	1684	31
***Chara braunii***	4436	146	-	-	4462	27
***Chlamydomonas reinhardtii***	312	160	-	-	324	171
***Chondrus crispus***	148	25	-	-	120	30
***Coffea canephora***	656	19	-	-	635	16
***Cyanidioschyzon merolae***	34	14	-	-	34	25
***Eutrema salsugineum***	637	82	-	-	699	48
***Glycine max***	1076	46	1076	46	998	38
***Gossypium raimondii***	1100	53	1097	53	-	-
***Helianthus annuus***	1859	149	-	-	1720	47
***Hordeum vulgare***	3124	92	-	-	1663	56
***Klebsormidium nitens***	153	48	-	-	120	38
***Malus domestica***	722	44	-	-	734	28
***Manihot esculenta***	873	39	869	39	-	-
***Marchantia polymorpha***	790	35	-	-	957	31
***Medicago truncatula***	1052	44	1018	43	958	35
***Nicotiana tabacum***	1830	57	3002	114	-	-
***Oryza sativa Japonica***	733	31	733	31	733	25
***Ostreococcus tauri***	39	16	-	-	45	22
***Phaseolus vulgaris***	684	34	-	-	664	29
***Physcomitrella patens***	510	23	509	23	428	19
***Populus trichocarpa***	826	170	826	170	751	29
***Rosa chinensis***	855	28	-	-	782	23
***Selaginella moellendorffii***	1303	61	-	-	1154	43
***Solanum lycopersicum***	977	49	-	-	1023	43
***Solanum tuberosum***	991	51	-	-	930	44
***Sorghum bicolor***	583	32	583	32	-	-
***Triticum aestivum***	12763	340	12945	337	-	-
***Vitis vinifera***	712	31	709	30	720	18
***Zea mays***	2817	57	2083	45	1949	36

### The PltRNAdb database

The Plant tRNA database (PltRNAdb) was created as a data resource for the tRNA genes of 256 plant species. PltRNAdb was developed using several programming languages, including MySQL, PHP, Perl, Python, D3 library, CSS, HTML, and JavaScript. On the PltRNAdb website, researchers can search, browse, visualize, BLAST, and download predicted tRNA genes. Using the links in the main bar of the homepage, researchers can switch between database pages, including database search, quick access, resources, general statistics, BLAST, and bulk download pages.

The PltRNAdb search page offers researchers the ability to dive deep into the database and retrieve tRNA data in two steps. The first step is to select the plant species and the second step is to select the nuclear, chloroplast, or mitochondrial genome and the tRNA type. The tRNA types include Ala, Gly, Pro, Thr, Val, Arg, Leu, Phe, Asn, Asp, Glu, His, Ile, Met/iMet, Tyr, Supres, Cys, Ser, Trp, SelCys, Gln, Lys, and Undet. The results are displayed on the new page with the available details of the tRNA genes. The results page is divided into two subsections. The first is used to display statistical plots of the identified tRNAs in the species searched. The second section contains details such as tRNA sequence ID, chromosome/scaffold accession number, sequence start and end within the chromosome/scaffold, tRNA sequence, tRNA secondary structure visualization, tRNA type, anticodon, intron start, intron end, score, and notes. The tRNA secondary structure button leads to a separate page with details of the selected tRNA, including tRNA secondary structure image, tRNA type, anticodon, tRNA length, upstream and downstream sequence, and tRNA sequence. The results can be downloaded using the Download button at the top of the Results page.

The general statistics page of PltRNAdb offers researchers the ability to take a close look at all available statistics for their selected species. Researchers can select plant species from the drop-down menu using the scientific name of the plant. Summary statistics for the selected species include statistical charts of identified tRNAs and the summary table for the nuclear genome. In addition, the statistics of chloroplast and mitochondrial tRNAs when available. On the Bulk Download page, researchers can download all data for selected plant species. They can download the data in different formats, including the FASTA file of tRNAs, the identified tRNA details in a tabular format, and the statistics file for each genome separately (nuclear, chloroplast, or mitochondrial genome) (S1 Fig).

BLASTN is embedded in PltRNAdb for tRNAs DNA sequence comparisons. BLASTN allows researchers to quickly align their sequence to the tRNA sequences of 256 plant species. Researchers can blast their FASTA sequence against one of the 256 plant species. The results table includes the subject ID, query ID, identity, length, mismatch, gaps, query start and end, subject start and end, E-value, and blast score (S2 Fig).

### Case study: *Arabidopsis thaliana* tRNA genes

In the present study, we select *Arabidopsis thaliana* to show the user how to navigate PltRNAdb. Due to the high quality of the genome sequence and annotation of *Arabidopsis thaliana*, this case study also serves as a comparison between the current findings and the genome annotation provided by NCBI. In PltRNAdb, 642, 33, and 36 tRNA genes were detected in the nuclear, chloroplast, and mitochondrial genomes of *Arabidopsis thaliana*, respectively. In the NCBI genome database, a total of 623 tRNA genes were found in the annotation of *Arabidopsis thaliana*. Based on the location of the tRNA genes in the genome sequence, the 623 NCBI tRNA genes were compared with the 642 tRNA genes identified in the current study. Of the 623 NCBI tRNA genes, 622 match the current finding and only one NCBI tRNA gene has no match. The 20 tRNA genes from the current finding that were not present in the NCBI tRNA genes were 2 Lys, 2 Leu, 2 Glu, 1 Tyr, 1 Cys, 1 Arg, 1 Met, 1 Asp, 2 undetermined, and 7 pseudo-genes. The common tRNA genes, the tRNA genes unique to NCBI, and the tRNA genes unique to the current analysis are listed in S3 Table.

**Fig 3:**
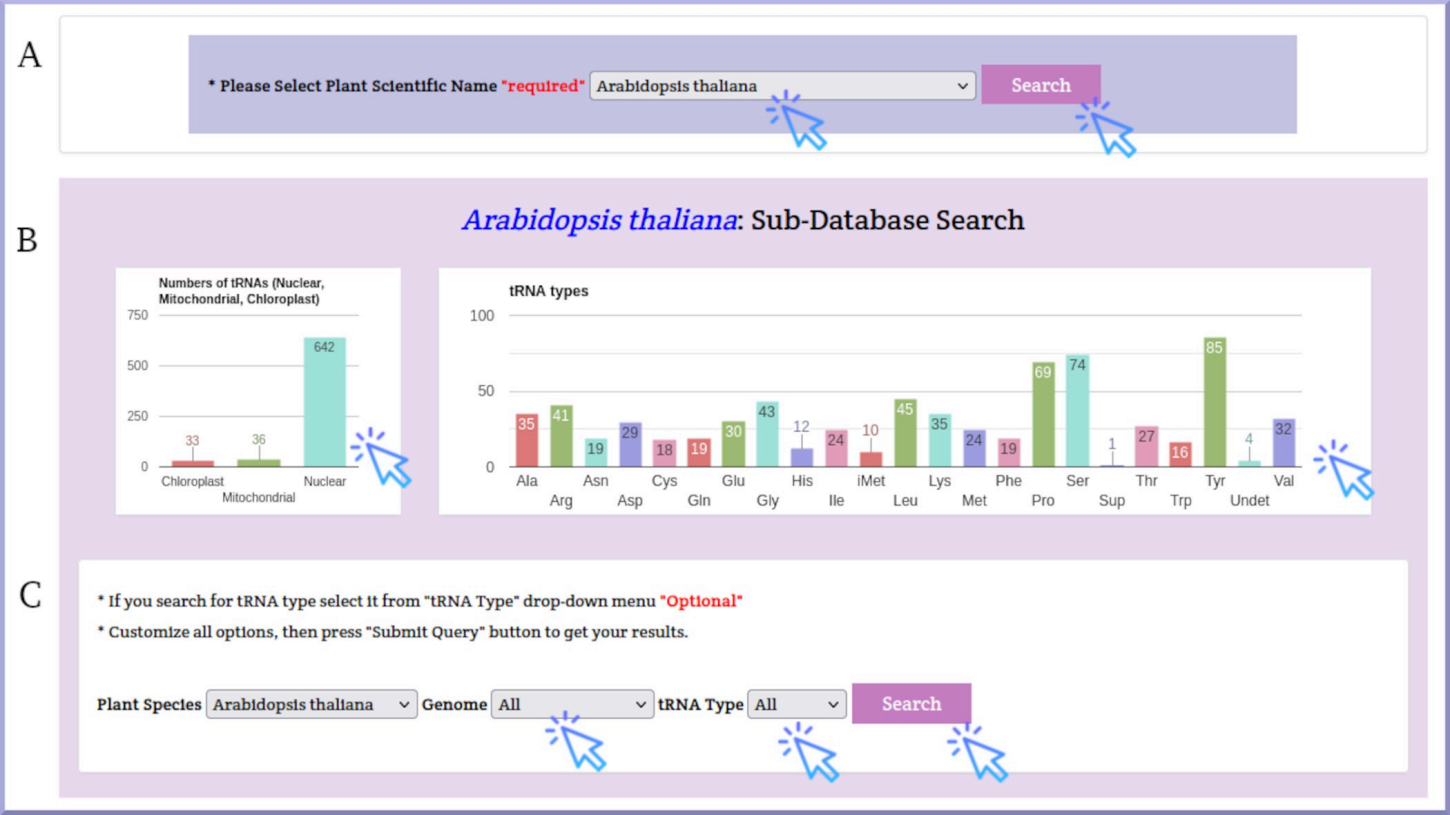
*Arabidopsis thaliana* search page as an example of PltRNAdb search. A) Select the plant species dropdown menu, B) bar charts represent statistics of all data available for *Arabidopsis thaliana* in PltRNAdb, C) Genome and tRNA dropdown menus for *Arabidopsis thaliana* search in PltRNAdb.

PltRNAdb includes searching, browsing, visualization, and downloading functionalities. The search page can be accessed via Database Search in the top bar of each page. First, select *Arabidopsis thaliana* from the plant species drop-down menu and click the Search button. The statistical charts of *Arabidopsis thaliana* tRNA genes are displayed on the same page. Second, select the nuclear, chloroplast, or mitochondrial genome and the tRNA type from the genome and tRNA Type drop-down menus (Fig 3).

**Fig 4:**
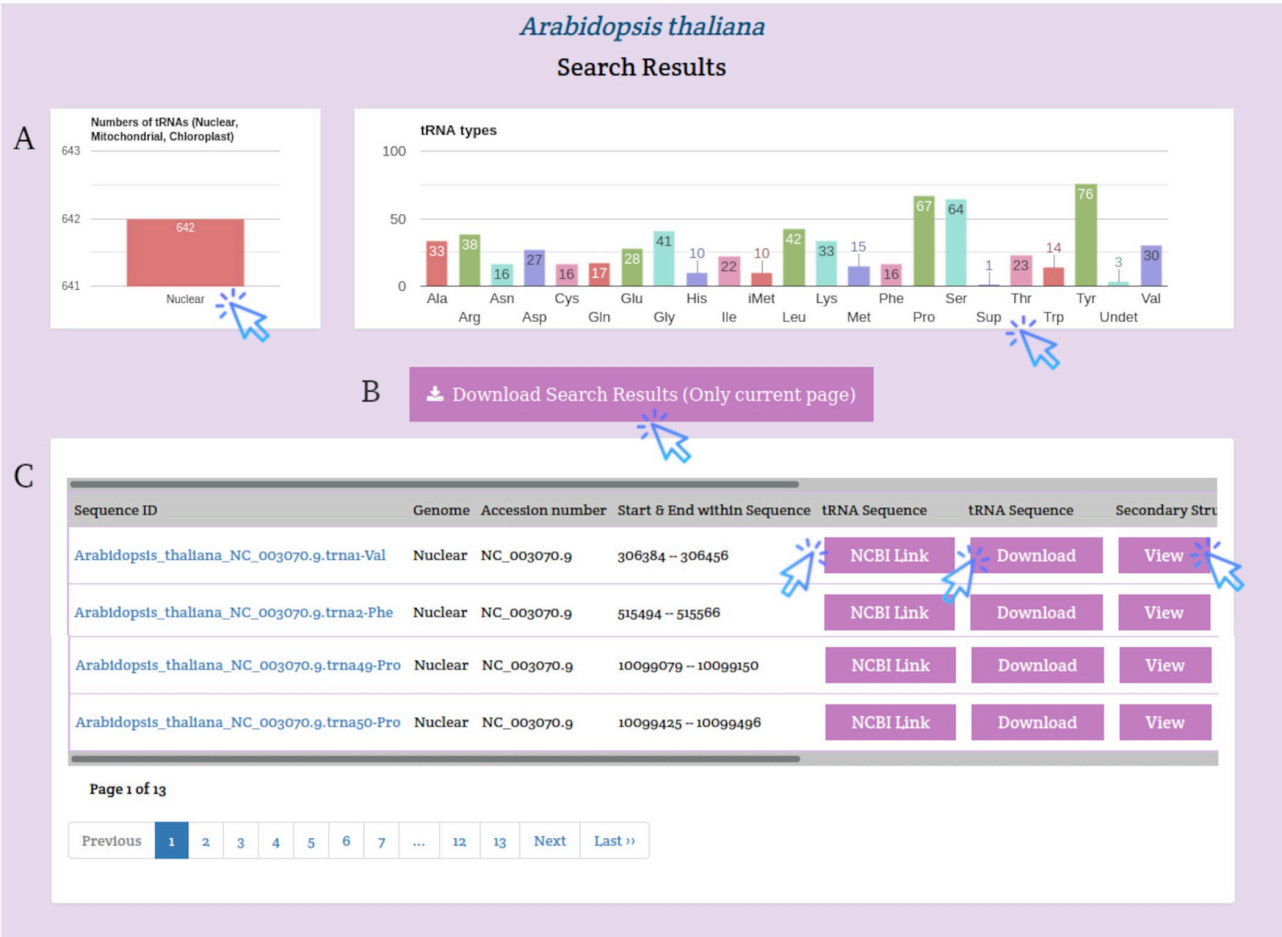
*Arabidopsis thaliana* search results. A) Visualize statistics subsection, B) button to download results, C) summary of search results with a hyperlink button for three separate pages to retrieve tRNA FASTA sequence from NCBI, download tRNA FASTA sequence from PltRNAdb, and retrieve details of the selected tRNA.

**Fig 5:**
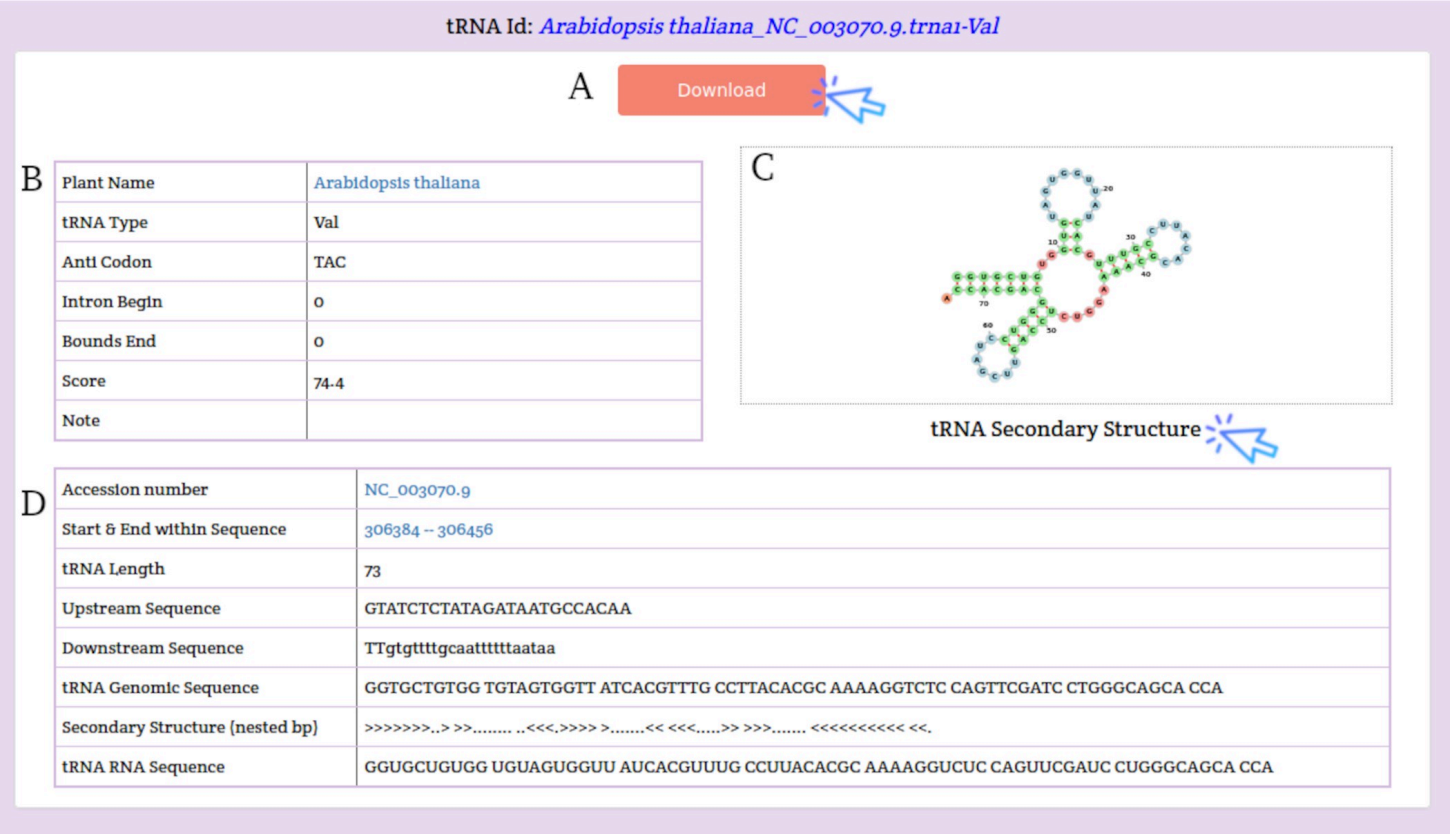
A view page of the selected tRNA contains A) a download button, B) summary statistics, C) visualization of tRNA secondary structure, D) tRNA start and end, tRNA length, tRNA sequence, and tRNA upstream and downstream sequences.

The search results are displayed on a separate page with the statistical charts of the tRNA genes subjected to the search parameters and a table with some details about the tRNA genes. Users can download the search results using the Download button at the top of the results table or download only the FASTA sequence for a tRNA gene by clicking the Download button in the tRNA Sequence column (Fig 4). Users can also access the details of the selected tRNA by clicking the View button in the Secondary Structure column. This page displays the details and image of the tRNA secondary structure of the selected tRNA as well as the download button (Fig 5).

**Fig 6:**
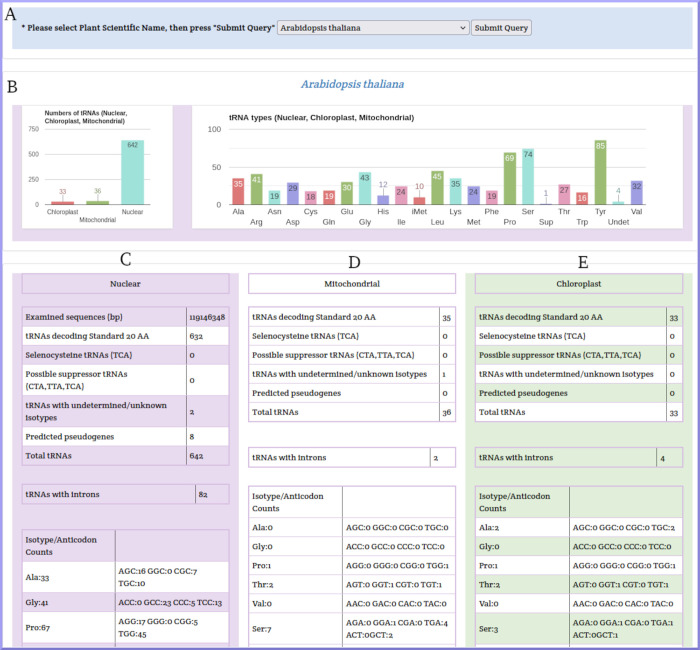
*Arabidopsis thaliana* general statistics page. A) Subsection for plant species selection, B) subsection for visualization of search results, C) statistics table of nuclear tRNAs, D) statistics table of mitochondrial tRNAs, E) statistics table of chloroplast tRNAs.

The general statistics page can be accessed by clicking the General Statistics button in the top bar of any page. This page is divided into three subsections. The first is for selecting *Arabidopsis thaliana* from the plant species dropdown menu. The second section contains the statistical charts of the total tRNA genes of *Arabidopsis thaliana* (nuclear, chloroplast, mitochondria) and a bar chart for the tRNA types. The third section contains statistical tables with nuclear, chloroplast, and mitochondrial values. The statistical tables include the total number of predicted tRNA genes, tRNAs decoding 20 standard amino acids, selenocysteine tRNAs (TCA), possible suppressor tRNAs, tRNAs with unknown isotypes, predicted pseudogenes, tRNAs with introns, and the number of isotypes/anticodons (Fig 6).

### Conclusion and future work

PltRNAdb is a database of tRNA genes, predicted by tRNAScan [8], for 256 plant species. Various tools and programming languages were used for visualize tRNA secondary structure, and build the database. PltRNAdb will be regularly updated with new annotated genomes and improve its tools to serve its purpose. Although PltRNAdb focuses on the prediction of tRNA genes in fully sequenced and annotated genomes, we plan to add a subsection for incomplete/unannotated plant genomes to the database to bring all available species together in one database. PltRNAdb will be an excellent resource for researchers interested in tRNAs research areas. We hope that PltRNAdb will improve our understanding of plant tRNAs and open the door to discovering the unknown regulatory roles of tRNAs in plant genomes.

## Supporting information

S1 Table List of plant names, NCBI taxid, assembly type and level, sequence representation, coverage, sequence category and accession numbers for all species examined. (XLSX)

S2 Table Total number of tRNAs and number of tRNAs with introns for all species unique to PltRNAdb. (XLSX)

S3 Table Comparison between tRNAs in PltRNAdb and tRNAs provided by NCBI for *Arabidopsis thaliana*. (XLS)

S1 Fig An example of the bulk download page. The first subsection is used to select the plant species, and the second subsection is used to select the type of data to download.
(PNG)

S2 Fig An example of the tRNA BLAST webservice. A) The BLAST interface webpage, B) The results webpage.
(JPG)
