# Supplementary figures and images for: PltRNAdb: Plant transfer RNA database
Source: PLoS One. 2022 May 23;17(5):e0268904. doi: 10.1371/journal.pone.0268904 (PMC9126412; doi:10.1371/journal.pone.0268904)

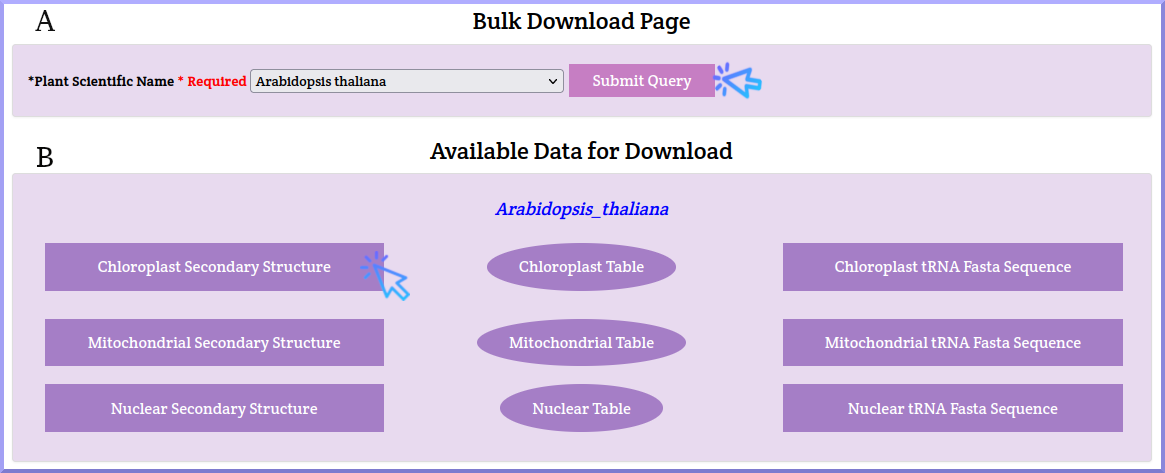

Supplement: S1 Fig — The first subsection is used to select the plant species, and the second subsection is used to select the type of data to download. (PNG) [file pone.0268904.s004.png]

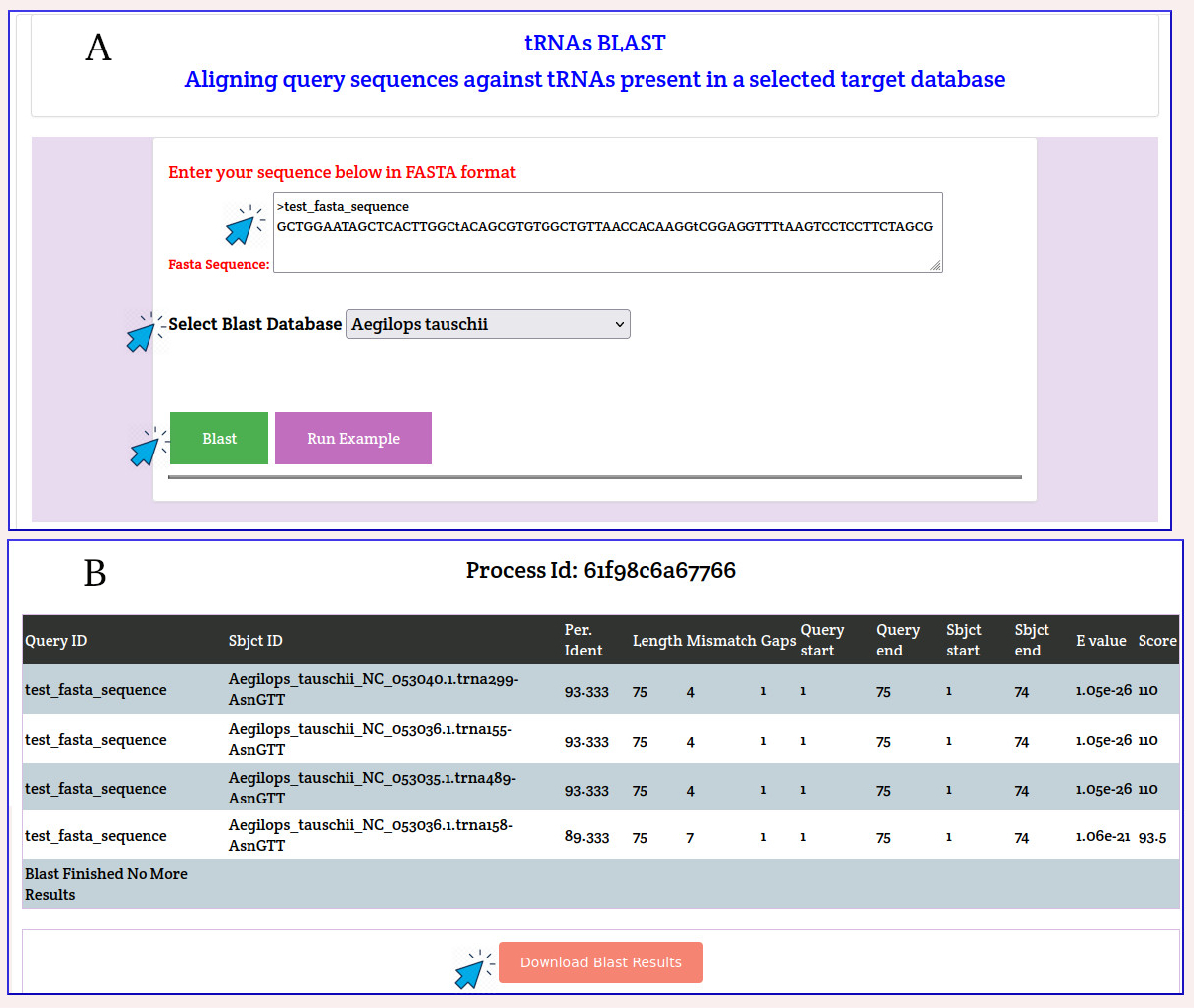

Supplement: S2 Fig — A) The BLAST interface webpage, B) The results webpage. (JPG) [file pone.0268904.s005.jpg]
